# CB_1_ and LPA_1_ Receptors Relationship in the Mouse Central Nervous System

**DOI:** 10.3389/fnmol.2019.00223

**Published:** 2019-09-19

**Authors:** Estíbaliz González de San Román, Iván Manuel, Catherine Ledent, Jerold Chun, Fernando Rodríguez de Fonseca, Guillermo Estivill-Torrús, Luis Javier Santín, Rafael Rodríguez Puertas

**Affiliations:** ^1^Department of Pharmacology, Faculty of Medicine and Nursing, University of the Basque Country (UPV/EHU), Leioa, Spain; ^2^Institut de Recherche Interdisciplinaire en Biologie Humaine et Moléculaire, Université Libre de Bruxelles, Brussels, Belgium; ^3^Sanford Burnham Prebys Medical Discovery Institute, La Jolla, CA, United States; ^4^Instituto de Investigación Biomédica de Málaga-IBIMA, Málaga, Spain, 5 Unidad de Gestión Clínica de Salud Mental, Málaga, Spain; ^5^Unidad de Gestión Clínica de Salud Mental, Hospital Regional Universitario de Málaga, Universidad de Málaga, Málaga, Spain; ^6^Unidad de Gestión Clínica de Neurociencias, Hospital Regional Universitario de Málaga, Málaga, Spain; ^7^Departamento de Psicobiología y Metodología de las Ciencias del Comportamiento, Universidad de Málaga, Málaga, Spain; ^8^Neurodegenerative Diseases, Biocruces Bizkaia Health Research Institute, Barakaldo, Spain

**Keywords:** neurolipids, lysophosphatidic acid, cannabinoids, GPCR, autoradiography, imaging mass spectrometry, brain

## Abstract

Neurolipids are a class of bioactive lipids that are produced locally through specific biosynthetic pathways in response to extracellular stimuli. Neurolipids are important endogenous regulators of neural cell proliferation, differentiation, oxidative stress, inflammation and apoptosis. Endocannabinoids (eCBs) and lysophosphatidic acid (LPA) are examples of this type of molecule and are involved in neuroprotection. The present study analyzes a possible relationship of the main receptor subtypes for both neurolipid systems that are present in the central nervous system, the CB_1_ and LPA_1_ receptors, by using brain slices from CB_1_ KO mice and LPA_1_-null mice. Receptor-mediated G protein activation and glycerophospholipid regulation of potential precursors of their endogenous neurotransmitters were measured by two different *in vitro* imaging techniques, functional autoradiography and imaging mass spectrometry (IMS), respectively. Possible crosstalk between CB_1_ and LPA_1_ receptors was identified in specific areas of the brain, such as the amygdala, where LPA_1_ receptor activity is upregulated in CB_1_ KO mice. More evidence of an interaction between both systems was that the CB_1_-mediated activity was clearly increased in the prefrontal cortex and cerebellum of LPA_1_-null mice. The eCB system was specifically over-activated in regions where LPA_1_ has an important signaling role during embryonic development. The modifications on phospholipids (PLs) observed in these genetically modified mice by using the IMS technique indicated the regulation of some of the PL precursors of both LPA and eCBs in specific brain areas. For example, phosphatidylcholine (PC) (36:1) was detected as a potential LPA precursor, and phosphatidylethanolamine (PE) (40:6) and PE (p18:0/22:6) as potential eCB precursors. The absence of the main cerebral receptors for LPA or eCB systems is able to induce modulation on the other at the levels of both signaling and synthesis of endogenous neurotransmitters, indicating adaptive responses between both systems during prenatal and/or postnatal development.

## Introduction

During past decades, lipid molecules present in the CNS were considered components of cell membranes and organelles, intermediary metabolites, or constituents of the energy reservoirs. Currently, it is known that neurolipids are bioactive molecules that can act as neurotransmitters with neuromodulatory properties. Studies analyzing lipid molecules related to signaling systems are clarifying the complex integration of different lipid-metabolic pathways with other physiological functions mediated by lipids. The involvement of lipids in physiological and pathological processes can now also be analyzed by the development of new and potent analytical tools and instruments that enable rapid, high-throughput lipidomic analyses of rodent and human brain tissue sections at anatomical resolution, such as IMS (Gonzalez de San Roman et al., 2017; Martinez-Gardeazabal et al., 2017).

Lipid mediators, synthesized from glycerophospholipids, are a class of bioactive lipids that are produced locally through specific biosynthetic pathways in response to extracellular stimuli. These lipid molecules, which hereafter we will call neurolipids, mediate neurotransmission as important endogenous regulators of multiple cerebral processes related to neuroprotective actions, such as neural cell proliferation, differentiation, response to oxidative stress, inflammation and apoptosis. Neurolipids are transported to extracellular compartments (by non-well-defined mechanisms), usually bind to specific GPCRs to trigger intracellular signaling and responses in target cells, and then are sequestered rapidly through enzymatic or non-enzymatic processes (Murakami, 2011). Representative members of this class of neurolipids are eCBs and the LP, LPA.

The first CB receptor cDNA was cloned in 1990 as the first GPCR for lipid mediators (Matsuda et al., 1990). Two CB subtypes have been cloned: CB_1_, the most abundant CB receptor subtype in the CNS, and CB_2_, which is more restricted to glial cells. However, the CB_2_ receptor appears to be expressed by some neurons, particularly under certain pathological conditions (Van Sickle et al., 2005; Viscomi et al., 2009; Garcia-Gutierrez and Manzanares, 2011; Navarrete et al., 2012). Moreover, there is evidence for other possible G protein-coupled CBs in the brain (Jarai et al., 1999; Di Marzo et al., 2000; Breivogel et al., 2001). Concerning the other above-mentioned LPs, the first high-affinity cognate cell surface receptor for LPA that was identified as the most abundant in the CNS was LPA_1_, but two additional receptors have been identified (LPA_2_ and LPA_3_). More recently, another three LPA receptors were identified, which were somewhat divergent receptors (LPA_4_, LPA_5_, LPA_6_) (reviewed in Choi et al., 2010; Yung et al., 2014). These two families of GPCRs, the CB and the LPA GPCRs, are phylogenetically related. CBs share 18–20% identity of the amino acid sequence with LPA receptors, in particular with LPA_1_, LPA_2_, and LPA_3_ (Chun et al., 1999).

Both LPA and eCBs are synthesized mainly by the cleavage of phospholipids (PLs), probably at the cell membrane. The distribution pattern of CB_1_ and CB_2_ receptors in the developing and adult brain is highly correlated with the expression of the DAGLα/β enzymes, responsible for 2-AG synthesis, suggesting that PL precursors would also be closely localized (Bisogno et al., 2003; Yoshida et al., 2006; Oudin et al., 2011). However, little is known about the specific lipid species that constitute the eCB precursors and particularly, about their specific localization in the brain (Lu and Mackie, 2016). Therefore, one of the aims of the present study is to identify these potential precursors through MALDI-IMS assays. This technique has enriched lipidomic studies due to the new dimensions added by IMS, i.e., the anatomical mapping of the molecules in tissue sections, a key piece of information to understand the physiological roles played by lipids present in the tissue (Caprioli et al., 1997; Jackson et al., 2005; Skraskova et al., 2015; Mohammadi et al., 2016).

Supporting the relationship between the eCB and LPA systems, it is relevant that the endogenous ligands for these systems are closely related, e.g., 2-AG can be metabolized to 2-arachidonoyl-LPA through the action of a monoacylglycerol kinase (Kanoh et al., 1986; Shim et al., 1989). The opposite direction of this phosphorylation reaction, i.e., dephosphorylation, also seems to be possible (Hiroyama and Takenawa, 1998; Nakane et al., 2002). Thus, it appears that 2-arachidonoyl-LPA and 2-AG could be mutually interconverted within a cell, and the direction of the reaction may even differ among the subcellular compartments within the cells and could be modified depending on the availability of substrates, such as ATP (Zhao and Abood, 2013). However, 2-arachidonoyl LPA would not directly interact with the cannabinoid CB_1_ receptor, and 2-AG, in turn, would not display affinity for the LPA_1_ receptor (Nakane et al., 2002). Moreover, structural analyses of the crystal structure of antagonist-bound LPA_1_ receptor predict a functional crosstalk with CB_1_ receptors (Chrencik et al., 2015).

During the last decade, the roles of the eCB and LPA systems in the regulation of neuronal progenitor proliferation and differentiation have been described, involving both systems in neurogenesis at the subventricular zone (Birgbauer and Chun, 2006; Galve-Roperh et al., 2006). However, LPA, through binding to the LPA_1_ receptor, acts only during the prenatal development of the brain (Hecht et al., 1996). In the meantime, eCBs, through binding to CB_1_ and CB_2_ receptors, regulate the migration of subventricular zone-derived neuroblasts in the postnatal brain (Oudin et al., 2011). Nevertheless, the eCB system may also control embryonic neuronal development and maturation (Diaz-Alonso et al., 2012).

In addition, both systems display relevant roles in the limbic brain areas and pathways that control emotional processes, such as the amygdala connections, where both systems are involved in the extinction of fear-conditioned responses (Marsicano et al., 2002; Tan et al., 2010; Pedraza et al., 2014). Furthermore, both systems seem to be involved in the control of cognitive processes linked to the hippocampus, such as spatial memory. maLPA_1_-null mice showed, through the LPA_1_ receptor-signaling pathway, long-term spatial memory impairment, but not short-term memory impairment (Castilla-Ortega et al., 2010).

On the other hand, direct hippocampal CB_1_ activation by high doses of exogenous or endogenous cannabinoid compounds induce characteristic long-term memory impairment (Puighermanal et al., 2009). In the same way, the genetic deletion of CB_1_ receptor induces neuronal loss in CA1 and CA3 areas of the hippocampus, which triggers a decline in cognitive functions (Bilkei-Gorzo et al., 2005).

In summary, both neurolipid systems are implicated in cortical development in the prenatal brain, in the extinction of fear-conditioned behavior in the amygdala, and in the modulation of long-term memory in the hippocampus, suggesting a convergence in the control of similar physiological functions in the CNS. In the present study we analyze the adaptive changes that are probably produced during mouse prenatal and postnatal development in the eCB and LPA neurolipid systems using both maLPA_1_-null and CB_1_ KO mice, to analyze the interactions and crosstalk between the CB_1_ and LPA_1_ mediated-activities and to identify the anatomical localization of the possible precursors of the endogenous ligands for both signaling systems. Technically, the study combines functional [^35^S]GTPγS autoradiography with MALDI-IMS to analyze consecutive brain slices.

## Materials and Methods

### Chemicals

[^35^S]GTPγS (initial specific activity 1250 Ci/mmol) was purchased from Perkin Elmer (Boston, MA, United States). WIN55212-2 was purchased from Tocris (Bristol, United Kingdom). Oleoyl-L-α-lysophosphatidic acid sodium salt (LPA), 2-mercaptobenzothiazole (MBT), DL-dithiothreitol (DTT), guanosine-5′-diphosphate (GDP), guanosine-5′-o-3-triphosphate and β-radiation sensitive Kodak Biomax MR films were acquired from Sigma-Aldrich (St. Louis, MO, United States). The [^14^C]-microscales used as standards in the autoradiographic experiments were purchased from Amersham Biosciences (Buckinghamshire, United Kingdom) and American Radiolabeled Chemicals (ARC, St. Louis, MO, United States). Finally, for the preparation of the different buffers, the treatment of slides, re-crystallization of the matrix and film development, several additional compounds supplied from different laboratories were used. All the compounds were of the highest commercially available quality for the necessity of the neurochemical studies.

### Animals and Tissue Preparation

maLPA_1_-null homozygous (*n* = 8) and wild-type 3-month-old males (*n* = 8) (on a mixed background C57Bl/6 × 129SW) were obtained from Hospital Carlos Haya, Málaga, Spain, as a result of a collaboration with Dr. Estivill-Torrús’s research group. The maLPA_1_-null (from Málaga variant of LPA_1_-null) mouse colony arose spontaneously from the initially reported LPA_1_-null mouse line (Contos et al., 2000) while crossing heterozygous foundational parents within their original mixed background since phenotypic variations have been described in almost complete perinatal viability and show a reduced VZ, altered neuronal markers, and increased cortical cell death that results in a loss of cortical layer cellularity in adults (Estivill-Torrus et al., 2008; Matas-Rico et al., 2008).

CB_1_-knockout mice are a strain maintained at the University of the Basque Country and were provided by C. Ledent of the University of Brussels (Belgium). For this study, 9 week-old male CB_1_-knockout (*n* = 5) and wild-type mice (*n* = 5) were used. The generation of mice lacking CB_1_ CBs was described previously (Ledent et al., 1999). To homogenize the genetic background of the mice, the first generation of heterozygous mice was bred for 30 generations on a CD1 background, with selection for the mutant CB_1_ gene at each generation. Mice were housed in a temperature (22°C) and humidity-controlled (65%) room with a 12:12-h light/dark cycle (light between 08:00 and 20:00 h), with food and water *ad libitum*. All procedures were performed in accordance with European animal research laws (European Communities Council Directives 86/609/EEC, 98/81/CEE and 2003/65/CE; Commission Recommendation 2007/526/EC) and the Spanish National Guidelines for Animal Experimentation and the Use of Genetically Modified Organisms (Real Decreto 1205/2005 and 178/2004; Ley 32/2007 and 9/2003). All the experimental protocols were approved by the Local Ethics Committee for Animal Research at the University of the Basque Country (CEIAB/21/2010/Rodriguez Puertas).

Mouse brains were quickly removed under anesthesia. Then, tissues were frozen on dry ice and kept at −80°C. The brains were cut on a Microm HM550 cryostat (Thermo, Germany) to obtain 20 μm sections that were mounted onto gelatin-coated slides, and these were stored at −20°C until use.

### [^35^S]GTPγS Binding Assay

The tissue sections were air-dried for 15 min then washed in HEPES-based buffer (50 mM HEPES, 100 mM NaCl, 3 mM MgCl_2_, and 0.2 mM EGTA, pH 7.4) for 30 min at 30°C in a water bath. The preincubation was repeated in new buffer. In a second step, the slides were incubated for 2 h at 30°C supplemented with 2 mM GDP, 1 mM DTT, adenosine deaminase (3 μ/l) and 0.04 nM [^35^S]GTPγS. The agonist-stimulated binding was measured under the same conditions in the presence of the specific GPCR agonists oleoyl-L-α-lysophosphatidic acid sodium salt; LPA (10^–5^ M) and WIN55212-2 (10^–5^ M). Non-specific binding was determined in the presence of 10 μM non-labeled GTPγS. Sections were washed twice in an ice-cold (4°C) HEPES buffer (50 mM, pH 7.4), dipped in distilled water, and air-dried. Sections were exposed to autoradiography film (Kodak Biomax MR) together with ^14^C standards for 48 h at 4°C in hermetically closed cassettes.

### Quantitative Image Analysis of Autoradiograms

Films were scanned and quantified by transforming the optical densities into nCi/g of tissue equivalent (nCi/g t.e.) using ImageJ software (NIH-IMAGE, Bethesda, MA, United States) (developed at the U.S. National Institutes of Health and freely available on https://imagej.nih.gov/ij/). The [^14^C] radioactive standards that were co-exposed with the tissue slides were used to calibrate the optical densities with the level of radioactivity labeled to the sections. Experimental data were analyzed by using the computer programs GraphPad Prism (v. 5.0, GraphPad) and Microsoft Excel. Data are expressed as the mean values ± SEM. The basal binding was calculated as nCi/g tissue equivalent, using the values provided in the ^14^C microscales. The agonist stimulation of [^35^S]GTPγS binding is expressed by the percentage of stimulation over the basal (%) in the presence of the different agonists, calculated as: (([^35^S]GTPγS agonist-stimulated binding) × 100/([^35^S]GTPγS basal binding)) − 100. The regions of interest (ROI) were defined from the scanned film in the case of the GTPγS autoradiography and directly from the image obtained from MALDI-IMS, using counter stained consecutive sections and a mouse brain atlas, without knowing the genotype of the mice and without taking into account the intensities of the rest of the animals. Differences between regions for each mouse genotype (wild type vs. transgenic) were analyzed by the unpaired two-tailed Student’s *t* test.

### Sample Preparation for MALDI-IMS

The original lipid composition and anatomical characteristics of the tissue must be well-preserved throughout the sample-preparation process (Schwartz et al., 2003). The fresh-frozen brains of the mice were cut on a Microm HM550 cryostat (Thermo, Germany) to obtain 20 μm sections and stored at −20°C until the moment of use.

Once the initial tissue preparation steps were completed, the matrix was deposited on the tissue surface prior to analysis by a sublimation process using the protocol described by Gonzalez de San Roman et al. (2017). For tissue sections mounted on glass slides, sublimation was performed using 300 mg of MBT, which was used as a chemical matrix, to control the deposition time and temperature (30 min, 140°C). Therefore, it was possible to control the thickness of the matrix layer and optimize the S/N ratio of the mass spectra, avoiding lipid migration on the tissue slice thanks to the lack of solvents using this sublimation procedure.

### Mass Spectrometer

A MALDI LTQ-XL-Orbitrap (Thermo Fisher, San Jose, CA) equipped with a nitrogen laser (λ = 337 nm, rep. rate = 60 Hz, spot size = 80 × 120 μm) was used for mass analysis. Thermo’s ImageQuest and Xcaliber software were used for MALDI-IMS data acquisition. The images were acquired in both negative and positive ion modes. The positive ion range was 500–1000 Da, with 10 laser shots per pixel at a laser fluence of 15 μJ. The negative ion range was 400–1100 Da, with 10 laser shots per pixel at a laser fluence of 15 μJ. The target plate stepping distance was set to 150 μm for both the *x*- and *y*-axes by the MSI image acquisition software. The mass resolution was approximately 100,000 laser shots in both positive and negative ion modes. The data were normalized using the TIC values to avoid potential displacement in the masses along the tissue that may be induced by experimental factors, including irregularities of the surface.

### Image and Spectral Analysis for MALDI-IMS

The MALDI-IMS technique was applied to localize and semi-quantitatively analyze the different lipid species present in WT and maLPA_1_-null mice in positive and negative ion detection modes. We employed optimized experimental conditions for lipid detection previously set up by our research group and others, including selection of the MBT chemical matrix and deposition mode by sublimation procedures (Astigarraga et al., 2008; Yang and Caprioli, 2011). First, IMS of sagittal mouse brain sections was performed using the positive ion detection mode, and approximately 300 mass peaks were detected in the mass range from 500 to 900 m/z. Then, the same slices of tissue were analyzed in negative ion detection mode using a mass range from 400 to 1100 m/z. A complete mass spectrum was obtained from each pixel along all the surface of each brain tissue slice, obtaining the slice images as a composition of the intensity color of each peak (molecule or m/z) at each pixel. Specifically, an ion of interest can be extracted from the spectra, and an image of that particular ion distribution in the tissue can be visualized. Once we had analyzed at least five sagittal brain slices samples from five different animals of  WT and another five samples of maLPA_1_-null mice, we selected different ROIs, such as hippocampus, cortex, cerebellum, cc, and striatum. The spectrum normalization was calculated using the TIC for each mass to exclude a number of noise spectra. Next, we compared WT and maLPA_1_-null using Origin^®^8 software.

In summary, the MALDI images were generated using ImageQuest software (Thermo Scientific). Each of the m/z values was plotted for signal intensity per pixel (mass spectrum) across a given area (tissue section). The m/z range of interest was normalized using the ratio of the TIC for each mass spectrum. The intensity reached by each mass spectrum (intensity of each peak, m/z value or molecule) was further calculated as a ratio of the peak with the highest intensity, and the average spectrum was calculated with OriginPro 8 software (Northampton, MA, United States). The most intense peak was considered 100%. Then, the two-tailed unpaired *t* test was performed for the comparison of two groups. The results were considered significant if *p* ≤ 0.05.

### Peak Assignment

Analysis of the lipid composition at CNS tissue samples from mice included in the present study was complex. A large number of different possible lipid species were detected, and some of them shared similar masses and were not detected and/or assigned to a specific lipid molecule by previous studies. Nevertheless, the assignment of lipid species was facilitated by the use of the Lipid MAPS database^1^ and different previous reports (Berry et al., 2011; Martinez-Gardeazabal et al., 2017), using a 5 ppm mass accuracy as the tolerance window for the assignment. The numbers (x:y) following the glycerolipid species denote the total length and the number of double bonds of the acyl chains, respectively, while the GlcCer species numbers correspond to the length and number of double bonds of the acyl chain added to those of the attached sphing-4-enine (d18:1) or sphinganine (d18:0) base.

## Results

### [^35^S]GTPγS Binding Assay of maLPA_1_-Null Mouse Brain Sections

**FIGURE 1:**
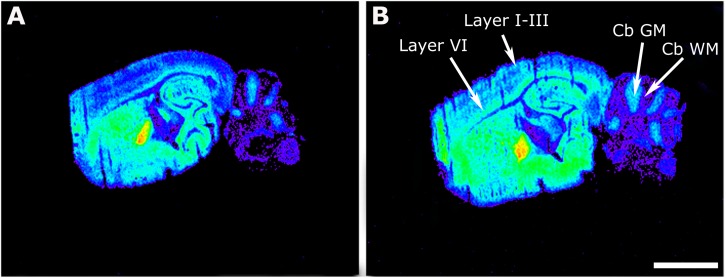
Representative autoradiograms of WT **(A)** and maLPA_1_-null mice **(B)** in sagittal sections that show [^35^S]GTPγS binding stimulated by WIN55212-2 (10^– 5^ M). Note the increase in the binding in maLPA_1_-null mice at different cortical layers (Layer I–III and Layer VI) and at the cerebellar gray matter (cb GM) and white matter (cb WM). Scale bar = 3 mm.

The [^35^S]GTPγS binding stimulated by WIN55212-2 was measured in brain slices of maLPA_1_-null and WT mice to localize and quantify the activity of CB_1_ CBs. The aim was to observe, in adult mice was the possible modulation of cannabinoid signaling that was induced by the lack of the LPA receptor subtype, LPA_1_, during the animal’s previous development. The basal activity (in the absence of agonist) of the G_i/o_-coupled GPCR in maLPA_1_-null mice was already shown in Gonzalez de San Roman et al. (2015). The G_i/o_-coupled CB_1_ receptor activity induced by the CB_1_ agonist WIN55212-2 was increased at the prefrontal cortex layers I–III (WT 212 ± 19% vs. maLPA_1_-null 369 ± 49%, *p* ≤ 0.05) and in layer VI (WT 284 ± 14% vs. maLPA_1_-null 396 ± 22%, *p* ≤ 0.05). The increase in the CB_1_ activity was also significant in the cerebellum gray matter (cb GM) of maLPA_1_-null mice compared to WT (WT 23 ± 15% vs. maLPA_1_-null 107 ± 13%, *p* ≤ 0.05) and cb WM (WT 678 ± 149% vs. maLPA_1_-null 1377 ± 207%, *p* ≤ 0.05). The globus pallidus, a nucleus with a high density of CB_1_ receptors in mice, also showed an increase in the activity of these receptors in maLPA_1_-null mice (WT 1705 ± 151% vs. maLPA_1_-null 2037 ± 82%), but this change was not statistically significant (Table 1 and Figure 1). The [^35^S]GTPγS binding stimulated by oleoyl-L-α-lysophosphatidic acid sodium salt (LPA) showed no stimulations in any brain area of the maLPA_1_-null mice, demonstrating the specificity of the experimental binding procedure and the right phenotype of the animals (Gonzalez de San Roman et al., 2015).

**TABLE 1:** [^35^S]GTPγS binding induced by WIN55212-2 (10 μM) in different areas of WT and maLPA_1_-null mice, calculated as percentage over the basal value.

	**Stimulation by WIN55212-2 (%)**		
**Brain region**	**WT**	**maLPA_1_-null**	***p*-value**
Amygdala	36250	23222	0.06
**Cerebellum**			
White matter	2.315	10713^∗^	0.001
Gray matter	679149	1377207^∗^	0.04
Corpus callosum	10820	15011	0.18
Striatum	25418	25523	0.75
**Frontal cortex**			
Layer I–III	21219	36949^∗^	0.05
Layer IV	18833	23614	0.27
Layer V	22325	28212	0.07
Layer VI	28414	39622^∗^	0.02
**Hippocampus**			
Dorsal CA1 radiata	37151	34138	0.69
Ventral CA3 radiata	28823	26612	0.51
Internal capsule	5316	3314	0.28
Globus pallidus	1705151	203782	0.21

*Data are mean ± SEM values, in percentage of stimulation over the basal (%). The *p*-values were calculated by the unpaired two-tailed Student’s *t* test. ^∗^*p* ≤ 0.05. WT(*n* = 8) and maLPA_1_null (*n* = 8).*

### [^35^S]GTPγS Binding Assay in CB_1_ KO Mouse Brain Sections

**FIGURE 2:**
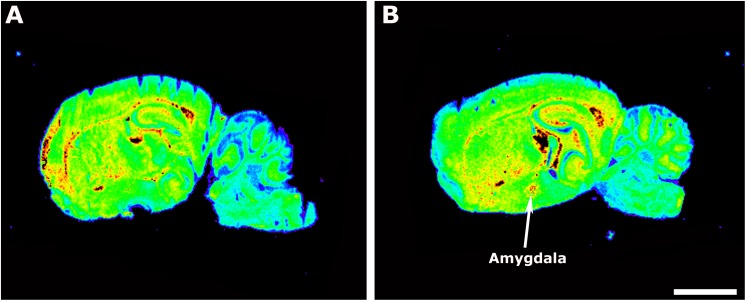
Autoradiographic images of sagittal sections from WT **(A)** and CB_1_ KO mice **(B)** showing [^35^S]GTPγS binding stimulated by LPA (10^– 5^ M). Note the increase in the binding at the amygdala in CB_1_ KO mice. Scale bar = 3 mm.

We compared the [^35^S]GTPγS binding stimulated by LPA in CB_1_ KO and WT mice to observe the consequences of the developmental absence of the main eCB receptor on the signaling through cerebral GPCRs for the LPA_1_ receptors. There were statistically significant changes that were restricted to the amygdala and showed increased [^35^S]GTPγS binding induced by LPA (WT −20 ± 4.3% vs. CB_1_ KO 32 ± 6.3%, *p* ≤ 0.05). The LPA_1_ receptor activity of CB_1_ KO mice was comparable to WT mice in all the other brain areas that were analyzed (Table 2 and Figure 2). The [^35^S]GTPγS binding stimulated by WIN55212-2 showed no stimulation in any brain area of the CB_1_ KO mice, demonstrating the specificity of the experimental binding procedure and the right phenotype of the animals (data not shown).

**TABLE 2:** [^35^S]GTPγS binding induced by LPA (10 μM) in different areas of WT and CB_1_ KO mouse brains, calculated as stimulation percentage over basal.

	**Stimulation by LPA (%)**		
**Brain region**	**WT**	**CB_1_ KO**	***p*-value**
Amygdala	−204.3	326.3^∗^	0.0008
**Cerebellum**			
White matter	22956	12446	0.2
Gray matter	506.5	5127	0.96
Corpus callosum	34372	32559	0.84
Striatum	219.3	245.5	0.8
**Frontal cortex**			
Layer I–III	6019	4621	0.6
Layer IV	5314	5727	0.62
Layer V	5011	6426	0.64
Layer VI	4815	6228	0.57
**Hippocampus**			
Dorsal CA1 radiata	4211	1823	0.38
Ventral CA3 radiata	1.413	145	0.44
Internal capsule	20341	21044	0.91
Globus pallidus	2.211	6242	0.16

*Data are mean ± SEM values, in percentage of stimulation over basal (%). SEM values, in percentage of stimulation over basal (%). The *p-*values were calculated by the unpaired two-tailed Student’s *t* test. ^∗^*p* ≤ 0.05. WT (*n* = 5) and CB_1_ KO (*n* = 5).*

### MALDI-IMS in maLPA_1_-Null Mouse Brain Sections

**FIGURE 3:**
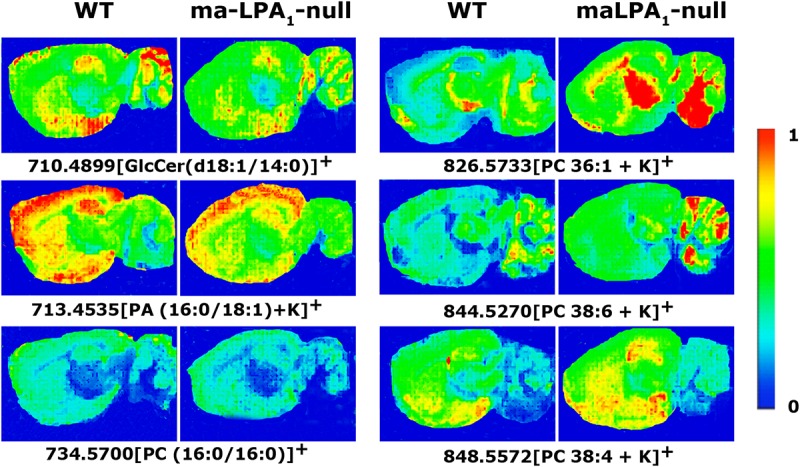
MALDI-IMS images representing different lipid species that showed statistically significant differences in the detection intensities between WT and maLPA_1_-null mice. The color images were obtained following the IMS conditions of 150 μm of spatial resolution, 10 shots per pixel at laser fluence of 15 μJ. The intensity of each lipid species is represented on the color scale, shown on the right side, as a ratio of the most abundant signal.

Some lipid species showed changes in positive ion detection mode in maLPA_1_-null mice compared to WT mice. Three lipid species, GlcCer (d18:1/14:0) + K^+^ in cortex (WT 14.5 ± 0.8% vs. maLPA_1_-null 9.3 ± 1.5%, *p* < 0.05) and striatum (WT 22.0 ± 1.3% vs. maLPA_1_-null 15.2 ± 2.3%, *p* ≤ 0.05), PA (16:0/18:1) + K^+^ in hippocampus (WT 46.5 ± 2.0% vs. maLPA_1_-null 37.2 ± 3.4%, not significant) and cortex (WT 47.5 ± 2.0% vs. maLPA_1_-null 37.3 ± 3.5%, *p* ≤ 0.05), and PC(32:0)^+^ in hippocampus (WT 27.3 ± 3.6% vs. maLPA_1_-null 17.9 ± 0.5%, *p* ≤ 0.05), were present at lower levels in maLPA_1_-null mice. By contrast, the intensity of the peaks for PC(36:1) + K^+^ in hippocampus (WT 16.3 ± 0.8% vs. maLPA_1_-null 20.4 ± 0.5%, *p* < 0.05) and cortex (WT 15.8 ± 0.7% vs. maLPA_1_-null 19.6 ± 1.5%, *p* ≤ 0.05), PC(38:4) + K^+^ in hippocampus (WT 8.4 ± 0.4% vs. maLPA_1_-null 11.3 ± 1.0%, *p* ≤ 0.05) and cortex (WT 10.3 ± 0.7% vs. maLPA_1_-null 13.5 ± 1.1%, *p* ≤ 0.05) and PC(38:6) + K^+^ in hippocampus (WT 12.0 ± 0.6% vs. maLPA_1_-null 15.7 ± 0.9%, *p* ≤ 0.05) was higher in the KO mice (Table 3 and Figure 3. See “*p*” values in table of Supplementary  Material).

**TABLE 3:** MALDI-IMS intensities (expressed as % of the most abundant peak) of molecular species of PC and PA that were found modified in positive ion detection mode in sagittal brain sections from WT (*n* = 5) compared to the maLPA_1_-null (*n* = 5).

		**Cortex**		**Hippocampus**		**Striatum**		**Corpus callosum**		**Cerebellum**	
**Assignment**	**m/z**	**WT**	**LPA_1_-null**	**WT**	**LPA_1_-null**	**WT**	**LPA_1_-null**	**WT**	**LPA_1_-null**	**WT**	**LPA_1_-null**
**GlcCer(d18:1/14:0) + K^+^**	**710.4899**	14.5 ± 0.8	9.3 ± 1.5^∗∗^	22.6 ± 1.0	19.1 ± 2.4	22.0 ± 1.3	15.2 ± 2.3^∗^	14 ± 0.9	11.1 ± 1.7	21.3 ± 2.3	21.1 ± 3.5
**PA(16:0/18:1) + K^+^**	**713.4535**	47.6 ± 2.0	37.3 ± 3.5^∗^	46.5 ± 2.0	37.2 ± 3.4^∗^	45.1 ± 2.2	35.3 ± 3.3^∗^	34.6 ± 2.9	28.9 ± 2.9	34.5 ± 1.1	32.2 ± 4.3
**PC(32:0)^+^**	**734.5700**	20.2 ± 3.2	25.3 ± 7.1	27.1 ± 3.6	17.9 ± 1.7^∗^	28.1 ± 3.3	26.7 ± 6.1	19.5 ± 3.0	20.9 ± 4.3	21.4 ± 2.2	22.7 ± 4.4
**PC(36:1) + K^+^**	**826.5733**	15.8 ± 0.7	19.6 ± 1.5^∗^	16.3 ± 0.8	20.4 ± 0.5^∗^	22.2 ± 1	26.2 ± 0.9^∗^	37.7 ± 1.1	42.0 ± 3.6	28.1 ± 0.8	28.2 ± 0.9
**PC(38:6) + K^+^**	**844.5270**	10.3 ± 0.7	13.5 ± 1.1^∗^	8.4 ± 0.4	11.3 ± 1.0^∗^	8.3 ± 0.6	10.3 ± 1.2	6.5 ± 0.5	8.5 ± 1.6	12.9 ± 1.1	16.1 ± 0.6^∗^
**PC(38:4) + K^+^**	**848.5572**	8.1 ± 0.9	8.7 ± 1.1	12 ± 0.6	15.7 ± 0.9^∗^	11.1 ± 0.6	13.2 ± 1.0	8.9 ± 0.4	11.1 ± 1.1	5.3 ± 0.2	6.4 ± 0.4^∗^

*Data are mean ± SEM values. The *p-*values were calculated by the unpaired two-tailed Student’s *t* test ^∗^*p***<** 0.05, ^∗∗^*p***<** 0.01.*

### MALDI-IMS in CB_1_ KO Mouse Brain Sections

**FIGURE 4:**
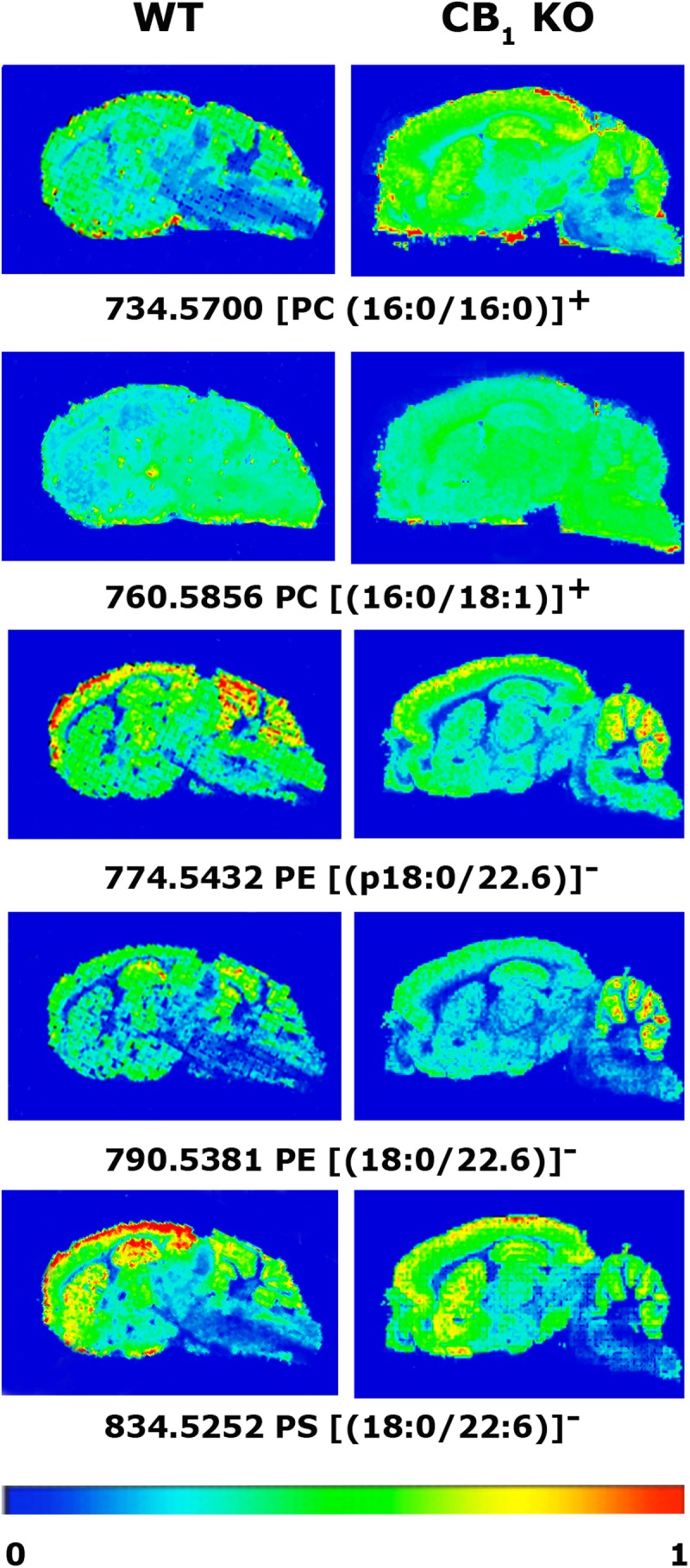
MALDI-IMS images representing the different lipid species that showed statistically significant differences in the detection intensities between WT and CB_1_ KO mice. The spectra were recorded with 150 μm spacing. Ten shots at each point were accumulated. Laser fluency was set at 15 μJ.

The results of the MALDI-IMS experiments using CNS slices from WT and CB_1_ KO mice were also performed in positive and negative ion detection modes. As has been described, we used an optimized protocol for the lipid detection. The analysis of IMS on sagittal mouse brain slices was performed in positive ion detection mode in a mass range from 500 to 1000 m/z and in negative ion detection mode from 400 to 1100 m/z. We selected the ROI and followed the steps described in the “Materials and Methods” section. The results indicated that some lipid species were modified in the brain of CB_1_ KO mice in both positive and negative ion detection modes. Some lipid species were present at a higher intensity than in WT mouse brain. These lipid species included PC(32:0)^+^ in hippocampus (WT 37.6 ± 3.6% vs. CB_1_ KO 65.3 ± 3.1%, *p* < 0.01) and cortex (WT 33.2 ± 3.7% vs. CB_1_ KO 54.7 ± 6.5%, *p* < 0.05) and PC(34:1)^+^ also in hippocampus (WT 48.6 ± 5.0% vs. CB_1_ KO 67.7 ± 3.5%, *p* < 0.05) and cortex (WT 51.2 ± 4.8% vs. CB_1_ KO 69.5 ± 4.5%, *p* < 0.05). Both PC molecules were increased in the frontal cortex, hippocampus and striatum but were not modified in the cerebellum of the CB_1_ KO mice. In contrast, the relative intensities of other lipid species were lower in CB_1_ KO mice than in WT mice in the hippocampus, frontal cortex and cerebellum but not modified in the striatum. These lipid species included PE(p18:0/22:6) in hippocampus (WT 3.4 ± 0.2% vs. CB_1_ KO 1.1 ± 0.6%, *p* < 0.05) and cortex (WT 3.7 ± 0.1% vs. CB_1_ KO 1.0 ± 0.6%, *p* < 0.05), PE(18:0/22:6)^–^ in hippocampus (WT 8.5 ± 0.3% vs. CB_1_ KO 4.4 ± 1.9%, *p* < 0.01), and phosphatidylserine (PS) (18:0/22:6)^–^ in cortex (WT 73.3 ± 9.5% vs. CB_1_ KO 46.9 ± 4.7%, *p* < 0.01) and striatum (WT 57.9 ± 4.8% vs. CB_1_ KO 40 ± 2.7%, *p* < 0.05) (Table 4 and Figure 4. See “*p*” values in table of Supplementary  Material).

**TABLE 4:** MALDI-IMS intensities (expressed as % of the most abundant peak) of molecular species of PC, PE, and PS in positive and negative ion detection modes that were modified in sagittal brain sections from WT (*n* = 5) compared to CB_1_ KO mice (*n* = 5).

		**Cortex**		**Hippocampus**		**Striatum**		**Cerebellum**	
**Assignment**	**Exp(m/z)**	**WT**	**CB_1_ KO**	**WT**	**CB_1_ KO**	**WT**	**CB_1_ KO**	**WT**	**CB_1_ KO**
**PC(32:0)^+^**	**736.5654**	33.23.7	54.76.5^∗^	37.63.6	65.43.1^∗∗^	31.44.1	49.36^∗^	42.37.5	51.44
**PC(34:1)^+^**	**760.5856**	51.24.8	69.54.5^∗^	48.65.0	67.73.5^∗^	51.75.2	67.75.1^∗^	65.27.9	72.34.2
**PE(p18:0/22:6)^–^**	**774.5432**	3.70.1	10.6^∗∗^	3.40.2	1.10.6^∗^	1.40.2	0.90.2	4.11.2	1.50.5
**PE(18:0/22:6)^–^**	**790.5381**	12.60.7	102.9	8.50.3	4.41.9^∗∗^	9.51.2	60.9	10.62	5.60.9^∗^
**PS(18:0/22:6)^–^**	**834.5252**	73.39.5	46.94.7^∗∗^	53.94.5	44.94.3	57.94.8	402.7^∗^	41.17.7	32.62.4

*Data are mean ± SEM values. The *p*-values were calculated by the unpaired two-tailed Student’s *t* test ^∗^*p* ≤ 0.05, ^∗∗^*p* ≤ 0.01.*

## Discussion

The present study shows specific anatomical alterations of a few lipid species that are potential PL precursors of LPA or eCBs, analyzing simultaneously in the same animals the regulation of the signaling by their specific CB_1_ and LPA_1_ receptors in KO mice for each of these receptors, which are the respective predominant subtypes present in the CNS. The analyzed regions were selected attending to previously described brain areas showing high densities of LPA_1_ or CB_1_ receptors.

The analysis of the activity of CB_1_ receptors in maLPA_1_-null mice allowed us to detect a regulation mechanism in both in the frontal cortex and in the cerebellum. The absence of LPA_1_ in maLPA_1_-null mice results in a reduction of neural cells in layers II/III, V, and VI, increased apoptosis, alterations in the formation of the cerebral cortex and premature expression of neuronal markers (Estivill-Torrus et al., 2008). The eCB system is a critical component of frontal cortex physiology. The CB_1_ receptor is mainly expressed in layers II/III and V/VI (Lafourcade et al., 2007), same areas where there is a depletion of neurons in maLPA_1_-null mice. Therefore, the increased functional activity of CB_1_ receptors suggests a compensatory effect of the eCB system to balance the neuronal reduction. Furthermore, the cerebellum is another brain area where we detected an increase in CB_1_ receptor activity in maLPA_1_-null mice. Cannabinoids modulate the proliferation of cerebellar neuronal precursors via the CB_1_ receptor (Trazzi et al., 2010). Hence, the lack of the LPA_1_ receptor could lead to an adaptive regulation in CB_1_-mediated activity.

On the other hand, when the activity of LPA_1_ receptors was analyzed in CB_1_ KO mice, the LPA_1_ receptor activity was increased only in the amygdala compared with WT mice. The CB_1_ receptor plays an important role in processes mediated by the amygdala, such as emotional responses (Azad et al., 2004; Laviolette and Grace, 2006; Roche et al., 2007; Tan et al., 2010). The eCB system in the amygdala has also been implicated in pain modulation (Manning et al., 2003), fear conditioning (Marsicano et al., 2002), anxiety (Imperatore et al., 2015) and fear-conditioned learning (Llorente-Ovejero et al., 2017). The LPA_1_ receptor plays a crucial role in the extinction of fear conditioning (Pedraza et al., 2014) and has been associated with the mixed depressive-anxiety phenotype (Moreno-Fernandez et al., 2017). Our results suggest that the lack of the CB_1_ receptor leads to an increased LPA_1_ receptor activity in the amygdala, as a compensatory effect exerted by LPA signaling in this limbic area. Considering the present results, it is difficult to hypothesize why no other brain regions have been regulated in CB_1_ KO mice, this regional exclusivity to the amygdala emphasizes the already reported role of LPA_1_ receptors in fear extinction, that might be indicating a close physiological control in anxiety behavior and fear-conditioned learning together with CB_1_ receptors. However, other functions modulated by the eCB system, such as pain or movement control, are possibly not being compensated by LPA_1_ signaling.

The lack of LPA_1_ receptor expression during mouse development led to an increase in the activity of the CB_1_ receptor in the cortical area and cerebellum, and the lack of the CB_1_ receptor led to an increase in LPA_1_ receptor activity in the amygdala. Therefore, the absence of neurotransmissions mediated by CB_1_ or LPA_1_ receptors in each strain of knockout mice converged to a common compensatory mechanism between both systems in some specific brain areas (cortex, cerebellum and amygdala). The reported compensation would affect receptors coupled to other types of G proteins, however, as previously mentioned, we were only able to analyze those coupled to the G_*i/o*_ subtype. It is improbable that CB_1_ receptors play a role since previous results reported a similar distribution regarding the CB_1_ receptor autoradiographic densities compared to functional autoradiography stimulated by specific agonists such as the WIN55212-2 (Llorente-Ovejero et al., 2018). However, it has not been yet possible to get the distribution of LPA_1_ receptors.

Moreover, the co-regulation of eCBs and LPA must be yielded during the pre- and postnatal development of both strains of mice.

The mechanisms underlying the observed modulation of CB_1_ receptors in LPA_1_-null mice may involve an increase in the expression of CB_1_ receptors mediated by another subtype of LPA receptors coupled to G_*i/o*_ proteins (see Supplementary  Material).

The metabolic precursors of the endogenous agonists for both neurolipid systems, eCB and LPA, are PLs present at the cellular membranes. The metabolism of PLs such as PC, PS and PE is thought to be responsible for the synthesis of LPA and the eCBs AEA and 2-AG. Therefore, we also hypothesized that the identification of specific PL modifications in the brain of CB_1_- and LPA_1_-KO mice would help us to understand the molecular neuroscience of the interactions between both systems. Thus, the PL precursor localization was an important step to clarify the synthesis of these lipid mediators. The application of the MALDI-IMS technique to maLPA_1_-null and CB_1_ KO mice allowed us to determine the anatomical localization of each lipid species in brain tissue slices. Unlike classical neurotransmitters, eCBs and LPA are not stored in specific compartments of the cells, such as vesicles, but instead are produced mainly “on demand” by stimulus-dependent cleavage of membrane PL precursors (Schmid et al., 1990; Di Marzo et al., 1994; Sugiura et al., 1995; Aoki, 2004). However, little is known about which are the specific lipid species for the synthesis of the endogenous agonists LPA, AEA, and 2-AG, or about their anatomical localization within the brain.

The results obtained with the MALDI-IMS assay in maLPA_1_-null and WT mice showed differences in the intensity of some lipid species, mainly in the positive ion detection mode. The affected species were PA(16:0/18:1) + K^+^ and four different PCs, including PC(32:0), PC(36:1) + K^+^, PC(38:6) + K^+^, and PC(38:4) + K^+^, whose more probable acyl chains were assigned based on previous studies and were identified as 16:0, 18:0, 18:1, 20:4, and 22:6 (Sugiura et al., 2009; Berry et al., 2011; Martinez-Gardeazabal et al., 2017). Although much has been learned about the physiological roles of LPA through the analyses of LPA receptors, the mechanism by which LPA is produced remains unclear. At least two pathways have been postulated. In the first pathway, LPA would be produced from PL by the autotaxin enzyme. In the second pathway, PA would be generated from PL or diacylglycerol and then deacylated by different phospholipases, such as PLA_1_ or PLA_2_ (Aoki, 2004; Aoki et al., 2008). The predominant fatty acyl moieties identified for LPA in rat brain are 18:1 (46.9%), 18:0 (22.5%), and 16:0 (18.8%), although a relatively small, but significant, amount of a 20:4-containing species has also been detected (7.2%) (Das and Hajra, 1989; Sugiura et al., 1999). PC species with arachidonic acid as its acyl chain PC(38:4), which was significantly increased in maLPA_1_-null mice in the cerebellum, could be a precursor of LPA (20:4). This species could be converted to 2-AG through LPA-phosphatase and trigger the increase in CB_1_ receptor activity (Nakane et al., 2002). Moreover, PC(36:1) is significantly increased in most of the analyzed areas and tended to increase in the maLPA_1_-null mice in brain areas where LPA_1_ receptors are specifically distributed, suggesting that PC(36:1) is a potential precursor for LPA synthesis (Gonzalez de San Roman et al., 2015; Mihara et al., 2019).

Regarding the MALDI-IMS results in CB_1_ KO and corresponding WT mice, the ratio of some lipid species was changed compared to WT mice, in both positive and negative ion detection modes. The affected species were PCs, PEs and PSs. PC(32:0) and PC(34:1) were increased approximately 50% in CB_1_ KO mice in some areas, such as the hippocampus and frontal cortex. We assumed that changes in PC(32:0) and PC(34:1) indicated that the lack of the CB_1_ receptor led to an increase in the abundance of lipids with palmitic acid. Both PCs can contain palmitic acid at fatty acyl moieties, and the CB_1_ receptor is palmitoylated for efficient coupling to a specific subset of G proteins located on membrane lipid rafts with a high concentration of saturated fatty acids (Oddi et al., 2012).

The synthesis of AEA and 2-AG could be produced by the cleavage or hydrolysis of membrane PL precursors. For the synthesis of 2-AG, at least three pathways have been proposed (Higgs and Glomset, 1994; Nakane et al., 2002; Kano et al., 2009). The synthesis of AEA seems to involve the enzymatic hydrolysis of the corresponding *N*-acyl-phosphatidylethanolamines (NAPE) (reviewed in Schmid et al., 1990, 1996; Hansen et al., 1998). In view of our results, we infer that the observed decrease in PE species in CB_1_ KO compared with WT mice could be related to precursors of AEA, due to the requirement of PE molecules for AEA synthesis. This decrease may be a modulation of PEs due to the lack of the target, the CB_1_ receptor. In addition, the decrease in PS species could be derived from the PE diminution because PE is one of the sources for PS synthesis (Vance and Tasseva, 2013). However, no changes in AEA or 2-AG levels have been reported between CB_1_ KO and WT mice (Maccarrone et al., 2001). The undergoing neuro-adaptive metabolic changes could lead to an increase in the activity of the enzymes responsible for the production of AEA from PE in KO mice, but obtaining the same levels of AEA than in WT.

Globally, the absence of LPA_1_ or CB_1_ receptors is able to induce modulation on the other system at the levels of both signaling and synthesis of endogenous neurotransmitters, suggesting adaptive responses between both systems during prenatal and/or postnatal development. Future goals would be to further clarify the relationship between the cannabinoid and lysophosphatidic systems as neurolipid systems with potential neuroprotective actions to identify new therapeutic treatments that could be developed for neurodegenerative diseases, such as Alzheimer’s disease.

## Ethics Statement

All procedures were performed in accordance with the European animal research laws (European Communities Council Directives 86/609/EEC, 98/81/CEE, and 2003/65/CE; Commission Recommendation 2007/526/EC) and the Spanish National Guidelines for Animal Experimentation and the Use of Genetically Modified Organisms (Real Decreto 1205/2005 and 178/2004; Ley 32/2007 and 9/2003). All the experimental protocols were approved by the Local Ethics Committee for Animal Research at the University of the Basque Country (CEIAB/21/2010/Rodriguez Puertas).

## Author Contributions

EG, IM, and RP contributed to the conception, design, and experiments of the study, performed the statistical analysis, and wrote the manuscript. CL provided and characterized the CB_1_ KO mice. JC, FR, GE-T, and LS provided and characterized the LPA_1_ null mice. GE-T contributed to the discussion of the manuscript. All authors contributed to the manuscript revision, read, and approved the submitted version.

## Conflict of Interest Statement

The authors declare that the research was conducted in the absence of any commercial or financial relationships that could be construed as a potential conflict of interest.

## Footnotes

1 http://www.lipidmaps.org/

## Abbreviations

2-AG: 2-araquidonoil glycerol
AEA: anandamide
cb WM: cerebellum white matter
CB: cannabinoid receptor
cc: corpus callosum
Cx layer VI: frontal cortex layer VI
eCB: endocannabinoid
GlcCer: glucosylceramide
GPCR: G protein-coupled receptor
ic: internal capsule
LP: lysophospholipid
LPA: lysophosphatidic acid
MALDI-IMS: matrix-assisted laser desorption/ionization imaging mass spectrometry
PA: phosphatidic acid
PC: phosphatidylcholine
PE: phosphatidylethanolamine
ROI: region of interest
TIC: total ion current
VZ: ventricular zone.

## References

[B1] Aoki J. (2004). Mechanisms of lysophosphatidic acid production. *Semin. Cell Dev. Biol.* 15 477–489. 10.1016/j.semcdb.2004.05.001 15271293

[B2] Aoki J. Inoue A. Okudaira S. (2008). Two pathways for lysophosphatidic acid production. *Biochim. Biophys. Acta* 1781 513–518. 10.1016/j.bbalip.2008.06.005 18621144

[B3] Astigarraga E. Barreda-Gómez G. Lombardero L. Fresnedo O. Castaño F. Giralt M. T. (2008). Profiling and imaging of lipids on brain and liver tissue by matrix-assisted laser desorption/ionization mass spectrometry using 2-mercaptobenzothiazole as a matrix. *Anal. Chem.* 80 9105–9114. 10.1021/ac801662n 18959430

[B4] Azad S. C. Monory K. Marsicano G. Cravatt B. F. Lutz B. Zieglgansberger W. (2004). Circuitry for associative plasticity in the amygdala involves endocannabinoid signaling. *J. Neurosci.* 24 9953–9961. 10.1523/JNEUROSCI.2134-04.2004 15525780 PMC6730232

[B5] Berry K. A. Hankin J. A. Barkley R. M. Spraggins J. M. Caprioli R. M. Murphy R. C. (2011). MALDI imaging of lipid biochemistry in tissues by mass spectrometry. *Chem. Rev.* 111 6491–6512. 10.1021/cr200280p 21942646 PMC3199966

[B6] Bilkei-Gorzo A. Racz I. Valverde O. Otto M. Michel K. Sastre M. (2005). Early age-related cognitive impairment in mice lacking cannabinoid CB1 receptors. *Proc. Natl. Acad. Sci. U.S.A.* 102 15670–15675. 10.1073/pnas.0504640102 16221768 PMC1266095

[B7] Birgbauer E. Chun J. (2006). New developments in the biological functions of lysophospholipids. *Cell Mol. Life Sci.* 63 2695–2701. 10.1007/s00018-006-6155-y 16988788 PMC11136021

[B8] Bisogno T. Howell F. Williams G. Minassi A. Cascio M. G. Ligresti A. (2003). Cloning of the first sn1-DAG lipases points to the spatial and temporal regulation of endocannabinoid signaling in the brain. *J. Cell Biol.* 163 463–468. 10.1083/jcb.200305129 14610053 PMC2173631

[B9] Breivogel C. S. Griffin G. Di Marzo V. Martin B. R. (2001). Evidence for a new G protein-coupled cannabinoid receptor in mouse brain. *Mol. Pharmacol.* 60 155–163. 10.1124/mol.60.1.155 11408610

[B10] Caprioli R. M. Farmer T. B. Gile J. (1997). Molecular imaging of biological samples: localization of peptides and proteins using MALDI-TOF MS. *Anal. Chem.* 69 4751–4760. 10.1021/ac970888i 9406525

[B11] Castilla-Ortega E. Sanchez-Lopez J. Hoyo-Becerra C. Matas-Rico E. Zambrana-Infantes E. Chun J. (2010). Exploratory, anxiety and spatial memory impairments are dissociated in mice lacking the LPA1 receptor. *Neurobiol. Learn. Mem.* 94 73–82. 10.1016/j.nlm.2010.04.003 20388543 PMC3684252

[B12] Choi J. W. Herr D. R. Noguchi K. Yung Y. C. Lee C. W. Mutoh T. (2010). LPA receptors: subtypes and biological actions. *Annu. Rev. Pharmacol. Toxicol.* 50 157–186. 10.1146/annurev.pharmtox.010909.105753 20055701

[B13] Chrencik J. E. Roth C. B. Terakado M. Kurata H. Omi R. Kihara Y. (2015). Crystal structure of antagonist bound human lysophosphatidic acid receptor 1. *Cell* 161 1633–1643. 10.1016/j.cell.2015.06.002 26091040 PMC4476059

[B14] Chun J. Contos J. J. Munroe D. (1999). A growing family of receptor genes for lysophosphatidic acid (LPA) and other lysophospholipids (LPs). *Cell Biochem. Biophys.* 30 213–242. 10.1007/BF02738068 10356643

[B15] Contos J. J. Fukushima N. Weiner J. A. Kaushal D. Chun J. (2000). Requirement for the lpA1 lysophosphatidic acid receptor gene in normal suckling behavior. *Proc. Natl. Acad. Sci. U.S.A.* 97 13384–13389. 10.1073/pnas.97.24.13384 11087877 PMC27233

[B16] Das A. K. Hajra A. K. (1989). Quantification, characterization and fatty acid composition of lysophosphatidic acid in different rat tissues. *Lipids* 24 329–333. 10.1007/bf02535172 2755310

[B17] Di Marzo V. Breivogel C. S. Tao Q. Bridgen D. T. Razdan R. K. Zimmer A. M. (2000). Levels, metabolism, and pharmacological activity of anandamide in CB(1) cannabinoid receptor knockout mice: evidence for non-CB(1), non-CB(2) receptor-mediated actions of anandamide in mouse brain. *J. Neurochem.* 75 2434–2444. 10.1046/j.1471-4159.2000.0752434.x 11080195

[B18] Di Marzo V. Fontana A. Cadas H. Schinelli S. Cimino G. Schwartz J. C. (1994). Formation and inactivation of endogenous cannabinoid anandamide in central neurons. *Nature* 372 686–691. 10.1038/372686a0 7990962

[B19] Diaz-Alonso J. Guzman M. Galve-Roperh I. (2012). Endocannabinoids via CB(1) receptors act as neurogenic niche cues during cortical development. *Philos. Trans. R. Soc. Lond. B Biol. Sci.* 367 3229–3241. 10.1098/rstb.2011.0385 23108542 PMC3481527

[B20] Estivill-Torrus G. Llebrez-Zayas P. Matas-Rico E. Santin L. Pedraza C. De Diego I. (2008). Absence of LPA1 signaling results in defective cortical development. *Cereb. Cortex* 18 938–950. 10.1093/cercor/bhm132 17656621

[B21] Galve-Roperh I. Aguado T. Rueda D. Velasco G. Guzman M. (2006). Endocannabinoids: a new family of lipid mediators involved in the regulation of neural cell development. *Curr. Pharm. Des.* 12 2319–2325. 10.2174/138161206777585139 16787257

[B22] Garcia-Gutierrez M. S. Manzanares J. (2011). Overexpression of CB2 cannabinoid receptors decreased vulnerability to anxiety and impaired anxiolytic action of alprazolam in mice. *J. Psychopharmacol.* 25 111–120. 10.1177/0269881110379507 20837564

[B23] Gonzalez de San Roman E. Manuel I. Giralt M. T. Chun J. Estivill-Torrus G. Rodriguez (2015). Anatomical location of LPA1 activation and LPA phospholipid precursors in rodent and human brain. *J. Neurochem.* 134 471–485. 10.1111/jnc.13112 25857358 PMC4780441

[B24] Gonzalez de San Roman E. Manuel I. Giralt M. T. Ferrer I. Rodriguez-Puertas R. (2017). Imaging mass spectrometry (IMS) of cortical lipids from preclinical to severe stages of Alzheimer’s disease. *Biochim. Biophys. Acta* 1859 1604–1614. 10.1016/j.bbamem.2017.05.009 28527668

[B25] Hansen H. S. Lauritzen L. Moesgaard B. Strand A. M. Hansen H. H. (1998). Formation of N-acyl-phosphatidylethanolamines and N-acetylethanolamines: proposed role in neurotoxicity. *Biochem. Pharmacol.* 55 719–725. 9586943 10.1016/s0006-2952(97)00396-1

[B26] Hecht J. H. Weiner J. A. Post S. R. Chun J. (1996). Ventricular zone gene-1 (vzg-1) encodes a lysophosphatidic acid receptor expressed in neurogenic regions of the developing cerebral cortex. *J. Cell Biol.* 135 1071–1083. 10.1083/jcb.135.4.1071 8922387 PMC2133395

[B27] Higgs H. N. Glomset J. A. (1994). Identification of a phosphatidic acid-preferring phospholipase A1 from bovine brain and testis. *Proc. Natl. Acad. Sci. U.S.A.* 91 9574–9578. 10.1073/pnas.91.20.9574 7937808 PMC44855

[B28] Hiroyama M. Takenawa T. (1998). Purification and characterization of a lysophosphatidic acid-specific phosphatase. *Biochem. J.* 336(Pt 2), 483–489. 10.1042/bj3360483 9820827 PMC1219894

[B29] Imperatore R. Morello G. Luongo L. Taschler U. Romano R. De Gregorio D. (2015). Genetic deletion of monoacylglycerol lipase leads to impaired cannabinoid receptor CB(1)R signaling and anxiety-like behavior. *J. Neurochem.* 135 799–813. 10.1111/jnc.13267 26223500

[B30] Jackson S. N. Wang H. Y. Woods A. S. (2005). In situ structural characterization of phosphatidylcholines in brain tissue using MALDI-MS/MS. *J. Am. Soc. Mass Spectrom.* 16 2052–2056. 10.1016/j.jasms.2005.08.014 16253515

[B31] Jarai Z. Wagner J. A. Varga K. Lake K. D. Compton D. R. Martin B. R. (1999). Cannabinoid-induced mesenteric vasodilation through an endothelial site distinct from CB1 or CB2 receptors. *Proc. Natl. Acad. Sci. U.S.A.* 96 14136–14141. 10.1073/pnas.96.24.14136 10570211 PMC24203

[B32] Kano M. Ohno-Shosaku T. Hashimotodani Y. Uchigashima M. Watanabe M. (2009). Endocannabinoid-mediated control of synaptic transmission. *Physiol. Rev.* 89 309–380. 10.1152/physrev.00019.2008 19126760

[B33] Kanoh H. Iwata T. Ono T. Suzuki T. (1986). Immunological characterization of sn-1,2-diacylglycerol and sn-2-monoacylglycerol kinase from pig brain. *J. Biol. Chem.* 261 5597–5602. 3007514

[B34] Lafourcade M. Elezgarai I. Mato S. Bakiri Y. Grandes P. Manzoni O. J. (2007). Molecular components and functions of the endocannabinoid system in mouse prefrontal cortex. *PLoS One* 2:e709. 10.1371/journal.pone.0000709 17684555 PMC1933592

[B35] Laviolette S. R. Grace A. A. (2006). Cannabinoids potentiate emotional learning plasticity in neurons of the medial prefrontal cortex through basolateral amygdala inputs. *J. Neurosci.* 26 6458–6468. 10.1523/JNEUROSCI.0707-06.2006 16775133 PMC6674051

[B36] Ledent C. Valverde O. Cossu G. Petitet F. Aubert J. F. Beslot F. (1999). Unresponsiveness to cannabinoids and reduced addictive effects of opiates in CB1 receptor knockout mice. *Science* 283 401–404. 10.1126/science.283.5400.401 9888857

[B37] Llorente-Ovejero A. Manuel I. Giralt M. T. Rodríguez-Puertas R. (2017). Increase in cortical endocannabinoid signaling in a rat model of basal forebrain cholinergic dysfunction. *Neuroscience* 362 206–218. 10.1016/j.neuroscience.2017.08.008 28827178

[B38] Llorente-Ovejero A. Manuel I. Lombardero L. Giralt M. T. Ledent C. Giménez-Llort L. (2018). Endocannabinoid and muscarinic signaling crosstalk in the 3xtg-ad mouse model of alzheimer’s disease. *J. Alzheimers Dis.* 64 117–136. 10.3233/JAD-180137 29865071

[B39] Lu H. C. Mackie K. (2016). An introduction to the endogenous cannabinoid system. *Biol. Psychiatry* 79 516–525. 10.1016/j.biopsych.2015.07.028 26698193 PMC4789136

[B40] Maccarrone M. Attina M. Bari M. Cartoni A. Ledent C. Finazzi-Agro A. (2001). Anandamide degradation and N-acylethanolamines level in wild-type and CB1 cannabinoid receptor knockout mice of different ages. *J. Neurochem.* 78 339–348. 10.1046/j.1471-4159.2001.00413.x 11461969

[B41] Manning B. H. Martin W. J. Meng I. D. (2003). The rodent amygdala contributes to the production of cannabinoid-induced antinociception. *Neuroscience* 120 1157–1170. 10.1016/s0306-4522(03)00356-7 12927220

[B42] Marsicano G. Wotjak C. T. Azad S. C. Bisogno T. Rammes G. Cascio M. G. (2002). The endogenous cannabinoid system controls extinction of aversive memories. *Nature* 418 530–534. 10.1038/nature00839 12152079

[B43] Martinez-Gardeazabal J. Gonzalez de San Roman E. Moreno-Rodriguez M. Llorente-Ovejero A. Manuel I. Rodriguez-Puertas R. (2017). Lipid mapping of the rat brain for models of disease. *Biochim. Biophys. Acta* 1859 1548–1557. 10.1016/j.bbamem.2017.02.011 28235468

[B44] Matas-Rico E. Garcia-Diaz B. Llebrez-Zayas P. Lopez-Barroso D. Santin L. Pedraza C. (2008). Deletion of lysophosphatidic acid receptor LPA1 reduces neurogenesis in the mouse dentate gyrus. *Mol. Cell Neurosci.* 39 342–355. 10.1016/j.mcn.2008.07.014 18708146 PMC3667670

[B45] Matsuda L. A. Lolait S. J. Brownstein M. J. Young A. C. Bonner T. I. (1990). Structure of a cannabinoid receptor and functional expression of the cloned cDNA. *Nature* 346 561–564. 10.1038/346561a0 2165569

[B46] Mihara Y. Horikawa M. Sato S. Eto F. Hanada M. Banno T. (2019). Lysophosphatidic acid precursor levels decrease and an arachidonic acid-containing phosphatidylcholine level increases in the dorsal root ganglion of mice after peripheral nerve injury. *Neurosci. Lett.* 698 69–75. 10.1016/j.neulet.2018.12.035 30593874

[B47] Mohammadi A. S. Phan N. T. Fletcher J. S. Ewing A. G. (2016). Intact lipid imaging of mouse brain samples: MALDI, nanoparticle-laser desorption ionization, and 40 keV argon cluster secondary ion mass spectrometry. *Anal. Bioanal. Chem.* 408 6857–6868. 10.1007/s00216-016-9812-5 27549796 PMC5012256

[B48] Moreno-Fernandez R. D. Perez-Martin M. Castilla-Ortega E. Rosell Del Valle C. Garcia-Fernandez M. I. Chun J. (2017). maLPA1-null mice as an endophenotype of anxious depression. *Trans.l Psychiatry* 7:e1077. 10.1038/tp.2017.24 28375206 PMC5416683

[B49] Murakami M. (2011). Lipid mediators in life science. *Exp Anim* 60 7–20. 10.1538/expanim.60.7 21325748

[B50] Nakane S. Oka S. Arai S. Waku K. Ishima Y. Tokumura A. (2002). 2-Arachidonoyl-sn-glycero-3-phosphate, an arachidonic acid-containing lysophosphatidic acid: occurrence and rapid enzymatic conversion to 2-arachidonoyl-sn-glycerol, a cannabinoid receptor ligand, in rat brain. *Arch. Biochem. Biophys.* 402 51–58. 10.1016/S0003-9861(02)00038-3 12051682

[B51] Navarrete F. Perez-Ortiz J. M. Manzanares J. (2012). Cannabinoid CB(2) receptor-mediated regulation of impulsive-like behaviour in DBA/2 mice. *Br. J. Pharmacol.* 165 260–273. 10.1111/j.1476-5381.2011.01542 21671903 PMC3252982

[B52] Oddi S. Dainese E. Sandiford S. Fezza F. Lanuti M. Chiurchiu V. (2012). Effects of palmitoylation of Cys(415) in helix 8 of the CB(1) cannabinoid receptor on membrane localization and signalling. *Br. J. Pharmacol.* 165 2635–2651. 10.1111/j.1476-5381.2011.01658.x 21895628 PMC3423250

[B53] Oudin M. J. Gajendra S. Williams G. Hobbs C. Lalli G. Doherty P. (2011). Endocannabinoids regulate the migration of subventricular zone-derived neuroblasts in the postnatal brain. *J. Neurosci.* 31 4000–4011. 10.1523/JNEUROSCI.5483-10.2011 21411643 PMC6623539

[B54] Pedraza C. Sanchez-Lopez J. Castilla-Ortega E. Rosell-Valle C. Zambrana-Infantes E. Garcia-Fernandez M. (2014). Fear extinction and acute stress reactivity reveal a role of LPA receptor in regulating emotional-like behaviors. *Brain Struct. Funct.* 219 1659–1672. 10.1007/s00429-013-0592-9 23775489

[B55] Puighermanal E. Marsicano G. Busquets-Garcia A. Lutz B. Maldonado R. Ozaita A. (2009). Cannabinoid modulation of hippocampal long-term memory is mediated by mTOR signaling. *Nat. Neurosci.* 12 1152–1158. 10.1038/nn.2369 19648913

[B56] Roche M. O’Connor E. Diskin C. Finn D. P. (2007). The effect of CB(1) receptor antagonism in the right basolateral amygdala on conditioned fear and associated analgesia in rats. *Eur. J. Neurosci.* 26 2643–2653. 10.1111/j.1460-9568.2007.05861.x 17970731

[B57] Schmid H. H. Schmid P. C. Natarajan V. (1990). N-acylated glycerophospholipids and their derivatives. *Prog. Lipid Res.* 29 1–43. 10.1016/0163-7827(90)90004-5 2087478

[B58] Schmid H. H. Schmid P. C. Natarajan V. (1996). The N-acylation-phosphodiesterase pathway and cell signalling. *Chem. Phys. Lipids* 80 133–142. 10.1016/0009-3084(96)02554-6 8681424

[B59] Schwartz S. A. Reyzer M. L. Caprioli R. M. (2003). Direct tissue analysis using matrix-assisted laser desorption/ionization mass spectrometry: practical aspects of sample preparation. *J. Mass Spectrom.* 38 699–708. 10.1002/jms.505 12898649

[B60] Shim Y. H. Lin C. H. Strickland K. P. (1989). The purification and properties of monoacylglycerol kinase from bovine brain. *Biochem. Cell Biol.* 67 233–241. 10.1139/o89-035 2550036

[B61] Skraskova K. Khmelinskii A. Abdelmoula W. M. De Munter S. Baes M. McDonnell L. (2015). Precise anatomic localization of accumulated lipids in mfp2 deficient murine brains through automated registration of sims images to the allen brain atlas. *J. Am. Soc. Mass Spectrom.* 26 948–957. 10.1007/s13361-015-1146-6 25916600 PMC4422856

[B62] Sugiura T. Kondo S. Sukagawa A. Nakane S. Shinoda A. Itoh K. (1995). 2-Arachidonoylglycerol: a possible endogenous cannabinoid receptor ligand in brain. *Biochem. Biophys. Res. Commun.* 215 89–97. 7575630 10.1006/bbrc.1995.2437

[B63] Sugiura T. Nakane S. Kishimoto S. Waku K. Yoshioka Y. Tokumura A. (1999). Occurrence of lysophosphatidic acid and its alkyl ether-linked analog in rat brain and comparison of their biological activities toward cultured neural cells. *Biochim. Biophys. Acta* 1440 194–204. 10.1016/s1388-1981(99)00127-4 10521703

[B64] Sugiura Y. Konishi Y. Zaima N. Kajihara S. Nakanishi H. Taguchi R. (2009). Visualization of the cell-selective distribution of PUFA-containing phosphatidylcholines in mouse brain by imaging mass spectrometry. *J. Lipid Res.* 50 1776–1788. 10.1194/jlr.M900047-JLR200 19417221 PMC2724791

[B65] Tan H. Lauzon N. M. Bishop S. F. Bechard M. A. Laviolette S. R. (2010). Integrated cannabinoid CB1 receptor transmission within the amygdala-prefrontal cortical pathway modulates neuronal plasticity and emotional memory encoding. *Cereb. Cortex* 20 1486–1496. 10.1093/cercor/bhp210 19880592

[B66] Trazzi S. Steger M. Mitrugno V. M. Bartesaghi R. Ciani E. (2010). CB1 cannabinoid receptors increase neuronal precursor proliferation through AKT/glycogen synthase kinase-3beta/beta-catenin signaling. *J. Biol. Chem.* 285 10098–10109. 10.1074/jbc.M109.043711 20083607 PMC2843172

[B67] Van Sickle M. D. Duncan M. Kingsley P. J. Mouihate A. Urbani P. Mackie K. (2005). Identification and functional characterization of brainstem cannabinoid CB2 receptors. *Science* 310 329–332. 10.1126/science.1115740 16224028

[B68] Vance J. E. Tasseva G. (2013). Formation and function of phosphatidylserine and phosphatidylethanolamine in mammalian cells. *Biochim. Biophys. Acta* 1831 543–554. 10.1016/j.bbalip.2012.08.016 22960354

[B69] Viscomi M. T. Oddi S. Latini L. Pasquariello N. Florenzano F. Bernardi G. (2009). Selective CB2 receptor agonism protects central neurons from remote axotomy-induced apoptosis through the PI3K/Akt pathway. *J. Neurosci.* 29 4564–4570. 10.1523/JNEUROSCI.0786-09.2009 19357281 PMC6665736

[B70] Yang J. Caprioli R. M. (2011). Matrix sublimation/recrystallization for imaging proteins by mass spectrometry at high spatial resolution. *Anal. Chem.* 83 5728–5734. 10.1021/ac200998a 21639088 PMC3136623

[B71] Yoshida T. Fukaya M. Uchigashima M. Miura E. Kamiya H. Kano M. (2006). Localization of diacylglycerol lipase-alpha around postsynaptic spine suggests close proximity between production site of an endocannabinoid, 2-arachidonoyl-glycerol, and presynaptic cannabinoid CB1 receptor. *J. Neurosci.* 26 4740–4751. 10.1523/JNEUROSCI.0054-06.2006 16672646 PMC6674155

[B72] Yung Y. C. Stoddard N. C. Chun J. (2014). LPA receptor signaling: pharmacology, physiology, and pathophysiology. *J. Lipid Res.* 55 1192–1214. 10.1194/jlr.R046458 24643338 PMC4076099

[B73] Zhao P. Abood M. E. (2013). GPR55 and GPR35 and their relationship to cannabinoid and lysophospholipid receptors. *Life Sci.* 92 453–457. 10.1016/j.lfs.2012.06.039 22820167

