# Supplementary material for: CB1 and LPA1 Receptors Relationship in the Mouse Central Nervous System
Source: Front Mol Neurosci. 2019 Sep 19;12:223. doi: 10.3389/fnmol.2019.00223 (PMC6761275; doi:10.3389/fnmol.2019.00223)
Supplement: Supplementary file 1 [file Data_Sheet_1.docx]

**Suppl. Material 1.**

|  |  | p-value | | | | |
| --- | --- | --- | --- | --- | --- | --- |
| **Assigment** | **Exp** | **Cortex** | **Hippocampus** | **Striatum** | **Corpus callosum** | **Cerebellum** |
| **GlcCer(d18:1/14:0)+K^+^** | **710.48** | 0.01 | 0.05 | 0.03 | 0.19 | 0.97 |
| **PA(34:1)+ K^+^** | **713.45** | 0.05 | 0.17 | 0.04 | 0.22 | 0.61 |
| **PC(32:0)^+^** | **734.57** | 0.58 | 0.04 | 0.85 | 0.8 | 0.8 |
| **PC(36:1)+ K^+^** | **826.57** | 0.05 | 0.002 | 0.02 | 0.86 | 0.96 |
| **PC(38:6) + K^+^** | **844.52** | 0.04 | 0.03 | 0.18 | 0.07 | 0.05 |
| **PC(38:4) + K^+^** | **848.55** | 0.6 | 0.009 | 0.13 | 0.1 | 0.05 |

WT (n=5) compared to LPA_1_-null mice (n=5).

|  |  | **p-value** | | | |
| --- | --- | --- | --- | --- | --- |
| **Assigment** | **Exp** | **Cortex** | **Hippocampus** | **Striatum** | **Cerebellum** |
| **PC(32:0)^+^** | **734.56** | 0.03 | 0.0006 | 0.04 | 0.1 |
| **PC(34:1)^+^** | **760.58** | 0.02 | 0.01 | 0.03 | 0.4 |
| **PE(p18:0/22:6)^-^** | **774.54** | 0.004 | 0.02 | 0.5 | 0.07 |
| **PE(18:0/22:6)^-^** | **790.53** | 0.4 | 0.004 | 0.06 | 0.05 |
| **PS(18:0/22:6)^-^** | **834.52** | 0.04 | 0.2 | 0.02 | 0.3 |

WT (n=5) compared to CB_1_ KO mice (n=5).

**Suppl. Material 2.**

**CB_1_ receptor expression in cell cultures of the neural line Neuro2A and of hippocampus from LPA_1_-null mice**

**Objective**

To further explore the underlying mechanisms of the possible relationship between CB_1_ and LPA_1_ signaling, we measured the expression of cannabinoid CB_1_ receptors in cell cultures of the neural line Neuro2A, and in primary cultures from hippocampus of LPA_1_-null mice, which were treated with LPA and Ki (LPA receptors antagonist). The pertussis toxin, PTX (G_i/0_ protein blocker), was also used to verify that the effects were mediated by G_i/0_ proteins.

**Material and Methods**

MgCl (50mM) (Invitrogen), 5x reaction buffer (Promega), Oligo dt (Invitrogen), RT (Promega), Taq DNA polymerase (Invitrogen), 10x PCR buffer (Denville), Neurobasal (Gibco), PEN/Strep Glutamine (Gibco), 0,25% trypsin EDTA (Gibco), Advanced DMEM/F12 (Gibco), Trizol (Life Technologies), dichlorotrimethylsilane (Sigma-Aldrich), ethidium bromide (invitrogen), Tris-acetate-EDTA buffer (TAE), agarose (Sigma-Aldrich), Ki16425 (Selleckchem), LPA(18:1) (Sigma-Aldrich).

**Animals**

LPA_1_-null and WT P3 mouse pups were used in Jerold Chun’s laboratory at The Scripps Research Institute. The animals were kept under controlled light, temperature and humidity conditions (light and dark cycle of 12 hours, 22ºC and 65-70% relative humidity).

**Cell lines**

The neural line Neuro2A, facilitated by the research group of Jerold Chun, was used.

**Primary cultures**

Primary cultures of hippocampus of P3 mouse pups were performed as follows. First, the hippocampus was dissected in dissection medium (Hank's Balanced Salt Solution (HBSS), ascorbic acid, glucose, PEN / STREP) and collected in sterile Eppendorf tubes, once the hippocampus had been collected, the dissection buffer was drained, 1 ml of 0.25% trypsin was added and it was placed at 37° C 15 min. Then, the trypsin was drained, taking care not to lose the tissue, and dissociation medium (Minimum Essential Media or MEM, DNase and ascorbic acid) was added, it was crushed about 10 times during 45 sec. with glass pasteur pipettes (the tip was burnt before use so that it was not sharp and did not damage the cells), and covered with sterile dimethyldichlorosilane. The supernatant was collected and filtered with a falcon filter, once collected in Eppendorf tubes it was centrifuged 5 min. at 1200 rpm, and the pellet was resuspended in 4 ml of medium (Neurobasal, B27, ascorbic acid). Before culturing the cells, they were counted to obtain a final concentration of 1*10^5^ cells per well. The cells were incubated for two days and the treatment was started. They were treated with BSA, LPA (10 μM), LPA + Ki16425 (10 μM) (30 min before), LPA + pertussis toxin (10 μM) (30 min before), and incubated for 18 h.

**RNA extraction**

This method was used for the neuronal line Neuro2A. 500 μl of trizol was added per well, homogenized by syringe about 10 times and incubated 5 min at room temperature, the lysate was collected in Eppendorf tubes, and 200 μl of chloroform was added for about 15 sec and then stirred. They were centrifuged at 1200g for 5 min. at 4 ° C. New Eppendorf tubes were prepared with 500 μl of 2-isopropanol and the aqueous phase of trizol-chloroform was added to the new Eppendorf tubes that were shaken and incubated for 10 min at room temperature. They were centrifuged at 12000g for 10 min at 4 ° C. The supernatant was drained and 1 ml 75% EtOH (in DEPC) was added and then centrifuged at 7200g for 5 min at 4 ° C and the supernatant was drained while being careful not to discard the pellet, and dried for a few minutes and before adding RNase-free water, depending on the size of the pellet (10-50μl). The samples were frozen.

In the case of primary cultures, RNA extraction was carried out using an RNeasy mini kit (Qiagen) because the expected amount of RNA was low.

**Obtaining complementary DNA (cDNA)**

The cDNA was obtained from RNA, first the RNA concentration of each sample was measured using the Implen nanophotometer, and the cDNA was obtained. Each sample was prepared with 1 μl of oligo dt + 1 μg of RNA and H_2_O (up to 9 μl) and incubated at 70 ° C for 5 min. Each sample was mixed with 11 μl of master mix (5x buffer, 50mM MgCl, 2.5mM dNTP, RNase out, RT enzyme), with a final volume of 20 μl per tube.

These were incubated in the thermal cycler for 5 min at 25° C, for 1 hour at 42° C and then for 15 min at 70° C, to obtain the cDNA. This was used for the subsequent qRT-PCR.

**Quantitative qRT-PCR**

The quantification of CB_1_ receptor expression in the neuronal line Neuro2A and in primary cultures was carried out by qRT-PCR (Corbett Research Rotor-Gene 3000) with the following primers: primers (1) forward primer CB_1_, 5'-AGCAAGGACCTGAGACATGC-3'; reverse primer CB_1_, 5'-TGTTATTGGCGTGCTTGTGC-3'.

**Results**

The aim of this project was to observe the relationship between the lysophosphatidic acid system and the cannabinoid system to explore the mechanism underlying the modulation of the cannabinoid system by LPA receptors and in LPA_1_-null mice.

The first set of experiments consisted of treating the Neuro2A neuronal line with BSA, LPA (10μM) and LPA (10μM) + Ki16425 (10μM), administering the Ki16425, 30 min before the BSA and LPA. They were incubated for 18 hours at 37° C in a CO_2_ incubator and then the RNA extraction was carried out using the previously described protocol. The next step was to obtain cDNA and the qRT-PCR for the CB_1_ receptors.

In this first set of experiments, we wanted to analyze the expression of CB_1_ receptors in the Neuro2A cell line by treating neurons with LPA at two different concentrations in order to optimize the appropriate concentration and antagonizing the effect of LPA with Ki16425. For statistical purposes, it was decided to make 3 replicas of the treatments. It was observed that when the neurons were treated with Ki16425, there was a significant increase in CB_1_ expression (Figure 1), which indicated a possible modulatory effect on the cannabinoid system when LPA receptors were blocked. It was also observed that the LPA concentration of 10 μM showed lower standard error.

**
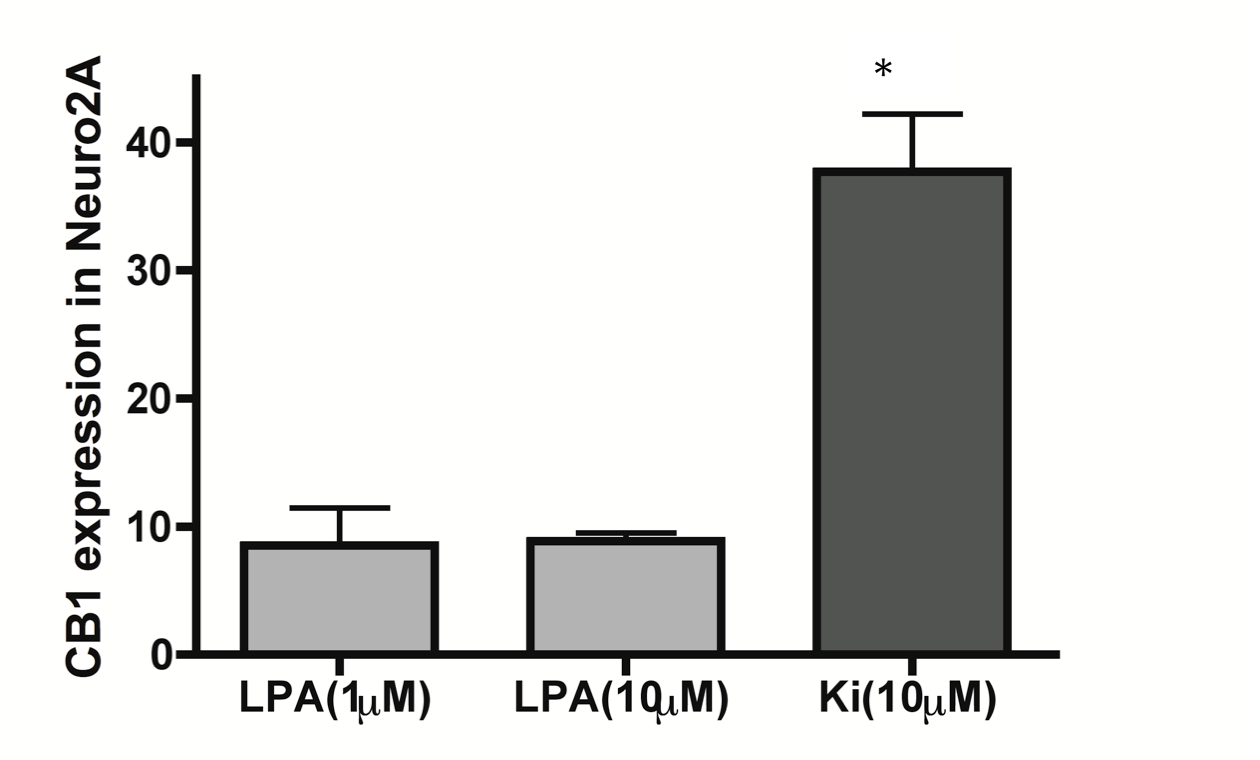
**

**Figure 1**. CB_1_ receptors expressed in neuronal cells Neuro2A (1*10^5^ cells/chamber), after treatment with LPA for 18h. qRT-PCR using the following primers: (1) forward primer CB_1_, 5'-AGCAAGGACCTGAGACATGC-3'; reverse primer CB_1_, 5'-TGTTATTGGCGTGCTTGTGC-3'. n= 3, *p≤ 0.05. The treatment with the specific LPA receptor antagonist, Ki16425, induces an up-regulation in the expression of CB_1_ receptors.

A modulation of the expression of cannabinoid CB_1_ receptors was observed after treating the cells with Ki16425 (10µM). Therefore, we decided to do a similar study that was also in primary hippocampal cultures of P3 of LPA_1_-null and WT mice. First the pups were genotyped. Then, the dissection of the hippocampus was carried out and the cells were cultured in 24-well plates. They were incubated for 48 hours and the treatment was carried out with BSA, LPA (10μM), LPA (10μM) + Ki16425 (10μM), LPA (10μM) + PTX (1nM), both the kKi16425 and PTX were administered 30 min. before LPA.

A significant increase in CB_1_ receptor expression was observed after treatment with LPA in the LPA_1_-null mice (Figure 2). However, when treated with Ki16425, which blocks the LPA receptors, the expression level was similar in LPA_1_-null and WT mice, indicating that other LPA receptor subtypes with an affinity for Ki16425 could be involved in these effects. These results were in line with those previously obtained in the Neuro2A cells. In LPA_1_-null mice there seems to be a greater expression of CB_1_ receptors by a possible modulation of the cannabinoid system when the LPA_1_ receptor has been deleted.

In this experiment, PTX treatment was also included to see if this effect was mediated through G_i/o_ proteins. In this case, the expression of CB_1_ was also similar in the LPA_1_-null and WT mice, indicating that GPCRs coupled to G_i/o_ proteins could be involved in this modulation of the CB_1_ receptors.

A similar study of the expression of LPA receptors in CB_1_ KO mice would also help to demonstrate the observed regulation in the amygdala of LPA activity in CB_1_ KO mice.


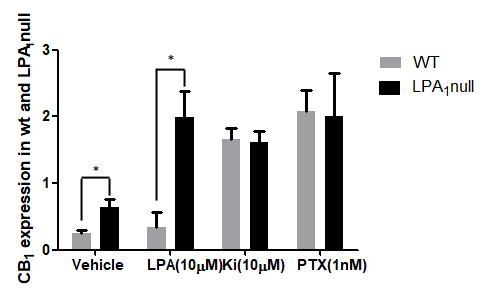


**Figure 2.** Expression of CB_1_ receptors in primary cell cultures from hippocampus of WT and LPA_1_ null mice (p3), treated with vehicle and for 18 h with LPA (10 µM), LPA + Ki16425 (10 µM) (30 min. before) or LPA + pertussis toxin (10 µM) (30 min. before). CB_1_ receptor expression was up-regulated after the treatment with LPA in LPA_1_ null mice. However, the expression was comparable to WT when treated with the LPA receptor antagonist, Ki16425. Furthermore, the pretreatment with PTX, also showed similar expression of CB_1_ receptors in WT and LPA_1_ null mice, indicating that the effect was mediated by GPCRs coupled to G_i/o_ proteins.


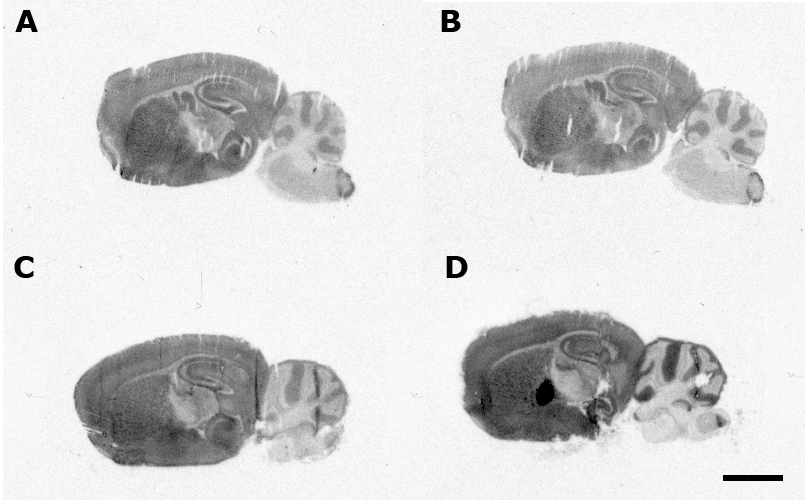


**Figure 3.** Representative autoradiograms of CB_1_ KO (A and B) and WT (C and D) mice in sagittal sections that show [^35^S] GTPγS basal binding (A and C) and stimulated by WIN55212-2 (10^-5^ M) (B and D). Note that WIN55212-2 (10^-5^ M) only produce stimulations in brain areas of WT animals coincident with CB_1_ receptor localization. Therefore, the contribution of other possible GPCRs with affinity for WIN55212-2 is negligible.
